# Is the speed of adjusting to environmental change condition dependent? An experiment with house mice (*Mus musculus*)

**DOI:** 10.1093/cz/zoae005

**Published:** 2024-03-07

**Authors:** Karem Lopez-Hervas, Neelam Porwal, Mathilde Delacoux, Alexandros Vezyrakis, Anja Guenther

**Affiliations:** RG Behavioural Ecology of Individual Differences, Max Planck Institute for Evolutionary Biology, 24306 Plön, Germany; RG Behavioural Ecology of Individual Differences, Max Planck Institute for Evolutionary Biology, 24306 Plön, Germany; Department of Evolutionary Biology, Faculty of Biology, Adam Mickiewicz University, Wieniawskiego 1, 61-712 Poznań, Poland; RG Behavioural Ecology of Individual Differences, Max Planck Institute for Evolutionary Biology, 24306 Plön, Germany; Department for Collective Behaviour, Max Planck Institute of Animal Behaviour, 78464 Constance, Germany; Centre for the Advanced Study of Collective Behaviour, University of Konstanz, 78464 Constance, Germany; RG Behavioural Ecology of Individual Differences, Max Planck Institute for Evolutionary Biology, 24306 Plön, Germany; Animal Ecology, Institute for Biochemistry and Biology, University of Potsdam, 14469 Potsdam, Germany; RG Behavioural Ecology of Individual Differences, Max Planck Institute for Evolutionary Biology, 24306 Plön, Germany

**Keywords:** animal personality, context dependence, environmental change, Match-mismatch, non-genetic inheritance, phenotypic flexibility, phenotypic plasticity

## Abstract

Environmental conditions change constantly either by anthropogenic perturbation or naturally across space and time. Often, a change in behavior is the first response to changing conditions. Behavioral flexibility can potentially improve an organism’s chances to survive and reproduce. Currently, we lack an understanding on the time-scale such behavioral adjustments need, how they actually affect reproduction and survival and whether behavioral adjustments are sufficient in keeping up with changing conditions. We used house mice (*Mus musculus*) to test whether personality and life-history traits can adjust to an experimentally induced food-switch flexibly in adulthood or by intergenerational plasticity, that is, adjustments only becoming visible in the offspring generation. Mice lived in 6 experimental populations of semi-natural environments either on high or standard quality food for 4 generations. We showed previously that high-quality food induced better conditions and a less risk-prone personality. Here, we tested whether the speed and/ or magnitude of adjustment shows condition-dependency and whether adjustments incur fitness effects. Life-history but not personality traits reacted flexibly to a food-switch, primarily by a direct reduction of reproduction and slowed-down growth. Offspring whose parents received a food-switch developed a more active stress-coping personality and gained weight at a slower rate compared with their respective controls. Furthermore, the modulation of most traits was condition-dependent, with animals previously fed with high-quality food showing stronger responses. Our study highlights that life-history and personality traits adjust at different speed toward environmental change, thus, highlighting the importance of the environment and the mode of response for evolutionary models.

In the face of environmental change, species, populations, and individuals within populations may be better able to adjust and survive by relying on phenotypic plasticity rather than on slower responses as genetic adaptations (DeWitt et al. 1998). Nowadays, it is generally assumed that adaptive plasticity induced by environmental factors plays an important role in evolution, allowing successful settling into new ecological niches or adaptation to changing environments (Bonduriansky et al. 2012).

Many behavioral traits are flexible by nature, allowing animals to respond quickly to changing environmental conditions (Duckworth 2009). Nevertheless, few studies investigate the evolutionary relevance of behavioral flexibility, probably because behavior was seen as inherently flexible for decades (Duckworth 2010; Duckworth et al. 2018). The last few decades, however, have shown that behavior is often limited in flexibility and differs consistently between individuals of the same species, age, or sex, and this is known as animal personality (Réale et al. 2007; Wolf and McNamara 2012). Animal personality can predict how individuals interact with their environment and can be adaptive if personality traits confer benefits in specific environments (Duckworth 2010; Duckworth et al. 2018). The expression of an adult personality type depends on the genetic background and is shaped throughout development by various environmental cues (Stamps and Groothuis 2010; Trillmich et al. 2018). For example, differences in predation pressure caused differences in average levels of boldness in a poecilid, *Brachyraphis episcopi* (Brown et al. 2005). Personality traits often vary continuously along a fast (e.g., bold, explorative, or aggressive)–slow (shy, non-explorative, and timid) axis. Often these personality traits correlate with fast–slow life-history traits such as early versus late reproduction. Under stable environmental conditions, such differences in average personality types often remain long-term or even life-long stable. When environmental conditions change, though, different personality types may react with varying levels of flexibility (Briffa et al. 2008; Carter et al. 2013). “Fast” personality types are predicted to be less behaviorally flexible than “slow” types in studies of captive animals (Benus et al. 1987; Coppens et al. 2010). Thus, these behaviorally flexible types may be better or faster in adjusting to changing environmental conditions (Dall et al. 2004).

Food availability and quality are known to impact personality and life-history traits. Zebra finches fed on high-quality food for example, develop a risk-averse, i.e., slow adult personality type (Krause et al. 2017). In contrast, individuals who consume low-quality food may have reduced performance and are often more prone to take risks in an attempt to gather resources (Moran et al. 2021). Along with the expressed behavioral average, food quality can affect behavioral variation, such as the spread of phenotypes, that is, among-individual variation, and behavioral consistency, that is, within-individual variation (Han and Dingemanse, 2017).

Many studies that have tested predictions regarding personality and flexibility come from laboratory settings. However, to understand mechanisms underlying fitness variation, predictions should be tested in the greater complexity of the natural environment (Archard and Braithwaite, 2010). Even though studies under natural conditions offer valuable insights, they often remain correlative and preclude experimental manipulations. In the present study, we use wild house mice (*Mus musculus domesticus*) living under semi-natural conditions to evaluate responses to an experimentally induced environmental change. In total, 6 experimental populations were fed with either standard quality (SQ) or high quality (HQ) food (HQ food contains more calories, protein, and fat, see Methods). In accordance with findings of a recent meta-analysis (Moran et al. 2021), we have previously found that the HQ condition induced a faster pace-of-life with an emphasis on early and fast reproduction whereas animals became less risk-prone within the first 3 generations compared with the SQ condition (Prabh et al. 2023). Higher reproductive rates were mainly driven by better body-condition and fast growth of HQ animals. Finding these effects on personality and life history raised the question of whether animals can rapidly adapt to fluctuating food quality.

We switched the food in one SQ and one HQ enclosure in adult animals of the third generation and in one enclosure each of the fourth generation. Thus, we created in the F4 generation the after experimental populations: SQ (control), SQ/HQ (food switch carried over from parents), SQ/HQ (within-generation food switch), HQ (control), HQ/SQ (food switch carried over from parents), and HQ/SQ (within-generation food switch). In all 6 populations, we tested animals for risk-taking in one forced and one voluntary situation. In the within-generation populations, we tested once before and once about one month after a food-switch. In addition to behaviors, we measure growth, reproduction, and survival.

Flexible adjustment to a changing environment is generally assumed to be costly, either in time that needs to be invested into sampling reliable cues (Dall and Cuthill, 1997) or by having to maintain highly costly neuronal machinery to allow flexible adjustment to changing environments (Snell-Rood 2013; McNamara and Barta 2020). Currently, we lack information on whether adult animals (within-generation food switches) are able to adjust flexibly within one month to a change in food. It may also be that subsets of individuals that cannot react flexibly to environmental change may die (i.e., selective disappearance, see Hämäläinen et al. 2014). Although flexibility involves reversible adjustments within an individual’s lifetime (Fagen 1982; Snell-Rood 2013), parental effects can transmit information about the environment from one generation to the next (intergenerational effects). Parental effects can shape behavior, morphology, physiology, and life-history traits of offspring (Piersma and Drent 2003). Both, flexibility and parental effects have been extensively discussed in terms of creating adaptive trait-changes (Mousseau and Fox 1998). The predictive adaptive response (PAR) hypothesis suggests that parental conditions can act as predictors for the offspring regarding their future environment and shape offspring development optimally (Bateson et al. 2014). Thus, under an adaptive scenario, we would expect animals being switched from SQ to HQ to reduce their risk-taking behavior while simultaneously increasing their reproduction and vice versa when animals are switched from HQ to SQ food (Engqvist and Reinhold 2016).

Three major hypotheses, generating in part different, potentially also non-adaptive predictions, may apply to our experiment. According to the silver-spoon hypothesis (Grafen 1990; Lindström 1999), good environmental conditions can have life-long positive effects on behavior, physiology, reproduction (e.g., litter- or clutch sizes), survival, and growth (Batzli 1986; Selman and Houston 1996; Reynolds et al. 2003; Jackson and Van Aarde 2004). In our experiment, animals in the HQ environment are in better condition (Porwal et al. 2023) and have more energy available. Therefore, we would expect animals either born in an HQ environment or exposed to HQ conditions to express higher flexibility after an environmental change, potentially demonstrating more pronounced or faster reactions than SQ animals (i.e., condition-dependent flexibility). This may carry-over to their offspring, manifesting in a stronger response (which may be adaptive or non-adaptive). Animals being switched from HQ to SQ food may experience reproductive decline when being faced with lower-than expected nutritional quality during breeding. Such effects may then carry-over to offspring, producing “thrifty-phenotypes.”

In contrast, the match-mismatch hypothesis (Cushing 1990), predicts that animals experiencing a mismatching adult environment from the environment they grew up in or in which their environment differs from that of their parents, will likely show a non-optimal phenotype and suffer fitness reductions, (O’Dea et al. 2016). Here, we created an experimental scenario in which the environment of the offspring differed from that in which parents grew up (SQ/HQ and HQ/SQ carried-over from parents). For our mice, this would suggest that offspring will neither behave as pure SQ or HQ animals whereas offspring developing in the same environment as their parents (matching conditions) will behave similarly to the previous generations. In line with predictions from the match-mismatch hypothesis, we would expect SQ/HQ and HQ/SQ environments to have reduced growth, fitness, or survival in comparison with their respective controls.

Lastly, it might also be that animals who are experiencing an unpredictable change in the environment may react with a general first-line response (adaptive or non-adaptive). In such a case, we might expect to see an increase or decrease in trait expression independent of the direction in which a food-switch occurred. A recent mathematical model for example suggests a general overshoot phenotype, that is, a phenotypic expression that is far away and/ or in an opposite direction from the optimum, to be likely when the environmental change is too strong for parental effects to produce optimal phenotypes (Hoyle and Ezard 2012). Taken together, we here aim to answer whether 1) adult mice can adjust behavior flexibly within one month after a switch of food type, 2) how such a food-switch affects reproduction and survival within the month directly after the food-switch, 3) how personality, growth, and reproduction of first-generation offspring are affected by a food-switch, and 4) whether the strength or speed of adjustment depends on the food quality regime, that is, on body condition (see Porwal et al. 2023).

## Materials and Methods

### Animals and housing

Semi-natural enclosures were established using descendants of wild house mice (*Mus musculus domesticus)* from the Cologne/Bonn region, Germany (50° 45ʹN-51° N, 6° 45ʹE-7° E). The founding generation for our experiment consisted of 160 individuals equally distributed across four enclosures with 20 males/females each (for details see Prabh et al. 2023).

Food and water were provided ad libitum at 9 feeding stations, temperature and light varied naturally but we prevented temperatures below 10 °C and light was artificially supplemented from 8am to 4pm. Wood chips and nesting materials were offered in addition to thirteen nesting boxes per enclosure. Two of the populations received standard food (SQ, Altromin 1324) containing 11% fat, 24% protein, and 65% carbohydrates whereas the other two received high-quality food (HQ, Altromin 1414), containing 22% fat, 28% protein, and 50% carbohydrates and a total of ~12% more kcal/kg.

Every month, populations were monitored by catching all individuals and marking new offspring > 10g with RFID subcutaneous transponders (ISO Transponders, PLanetID). Ear clips were sampled for later parentage assignment based on 17 unlinked microsatellite loci (Linnenbrink et al. 2013). Alleles were called using GeneMarker (V3.0.1) and parentages were assigned using Colony V 2.0.6.6 (Jones and Wang, 2010), assuming male and female polyandry, potential inbreeding, and applying full-likelihood. Whenever the next generation reached at least 80 marked offspring, the previous generation was removed to keep social density below the carrying capacity and avoid detrimental high densities.

### Experimental design

**Figure 1.**
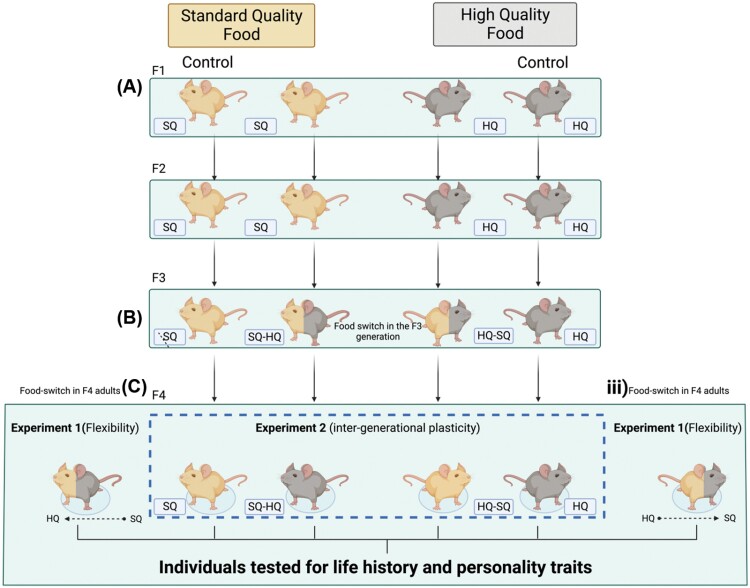
Experimental design: (A) Four populations were initially established receiving 2 different food qualities: standard (SQ) and high quality (HQ), for details see Prabh et al. (2023); (B) For the intergenerational food-switch experiment, we used a 2 × 2 full factorial design in the F3. Each one enclosure per food treatment received a food-switch whereas the other enclosure remained as control (no food-switch). In F4, adult animals (offspring from F3) were assessed for personality and life-history traits. (C) For the flexibility experiment, adult animals from the F4 control generation received a food-switch and were assessed both before and after the food-switch for life-history and personality traits. Mice in yellow, labeled ‘SQ’ under the paw, received SQ food and mice in gray labeled ‘HQ’ under the paw, received HQ food. Individuals that were exposed to a food switch are depicted with a start and half yellow, half grey, labeled ‘SQ-HQ’ or ‘HQ-SQ’. Created with BioRender.com

In the third generation, one enclosure per food type received a food-switch (Figure 1) and we investigated effects on behaviors and life-history traits in the offspring generation (inter-generational plasticity). In addition, we established 2 more enclosures (one SQ, one HQ) with F4 offspring of the control F3 enclosures and investigated immediate effects of a food-switch in adulthood (flexibility experiment) in parallel to intergenerational effects.

### Behavior

We assessed aspects of risk-taking using one voluntary exploration test (novel environment, NE) and one forced exploration test (open field, OF) in combination with assessing changes in core body temperature by infra-red thermography to assess individual measures of stress (see Table 1). Tests were repeated after 4 weeks to assess temporal consistency (i.e., repeatability). For the within-generation food-switch, the first test was done within one week before the food-switch and the second test was conducted after the food-switch. For catching, we distributed ~20 live traps across an enclosure. Each trapping session started in the evening hours close to sunset, lasted between 4 and 5 hours and traps were controlled every 10–20 min to reduce the handling time for mice as much as possible. The individuals were tested for NE and OF on separate days. A total of 162 individuals were tested for inter-generational effects of food-switch and 90 mice were tested for flexible adjustment to a food-switch.

**Table 1.** Overview of behavioral and physiological variables recorded throughout the study

Test	Category	Variable	Description of variable
Open field (OF) (5 min)	Behavioral	Distance covered	Active versus passive stress coping
	Behavioral	% Time in centre of OF	Behavioral measurements of stress experienced in the OF
	Physiological	ΔT	Physiological measurement of stress experienced in the OF
Novel environment (NE) (10 min)	Behavioral	Latency to leave trap	Proneness to explore versus stay in hiding. Mice that never left the trap scored 600s
	Behavioral	# of trips	Number of exploration bouts taken
	Behavioral	Exploration time	Time spent actively exploring

### Open field (OF)

Forced exploration was recorded (TSE Systems GmbH, Germany) for 5 min in a brightly illuminated and hence stressful OF (60 × 60 cm).

### Infrared-thermography

Directly before and after the OF, 3 infrared-photos (IR-Thermometry, FLIR T860) focusing on the eye of the mouse at a distance of ~40 cm from the live-trap were taken to assess the physiological stress–response (Lecorps et al. 2016). The body core temperature increases during a stressful situation and this increase can show consistent individual differences and reflects the physiological underpinning of the behavioral stress response seen in the OF (Lecorps et al. 2016). We calculated the average of the 3 photos at each time point and the change of temperature during the OF test Δ*T* = *T*_after_—*T*_before_ using the FLIR tools software.

### Novel environment (NE)

Recording of voluntary exploration behavior was directly conducted within semi-natural enclosures by inserting the mice in live-traps into a transparent Macrolon cage (Techniplast) (40.5 × 28.0 × 50.0 cm) containing bedding material with 3 unknown objects. Mice exploration was recorded under red light for 10 min (Panasonic Full HD camera 10 MP HC-V 180).

### Life-history

At each of the monthly monitorings, every individual was weighed and females were checked for reproductive activity, that is, being visibly pregnant and/ or lactating. Offspring weighing more than 10 g were individually marked with an RFID transponder.

To test how body mass was affected by a food-switch in adulthood, the change of body mass from the monitoring before the food-switch until the monitoring in which the food was switched was compared with to the mass change between the food-switch and the next monitoring. To assess inter-generational effects of a food-switch, the body mass development of all individuals was scored between the age of month 2 (i.e., being independent, see Prabh et al. 2023) and month 5.

To assess potential effects on reproduction immediately after a food-switch, we compared the percentage of adult females being reproductively active one month after the food-switch between both experimental populations (flexibility experiment). Likewise, we scored the percentage of reproductively active females between months 2 and 5 in the inter-generational part of the experiment.

To estimate immediate effects on survival, we counted the number of surviving individuals from one month before the food-switch until 1 month after the food-switch. To assess inter-generational effects, we counted the number of dead mice at each monthly monitoring over the 6 months.

### Statistical analyses

#### Behavior

All statistical analyses were performed using the free software R (R version 4.1.3, R Core Team 2023). We calculated mixed effects models using the function nlme::lme (Pinheiro and Bates 2023) for the distance covered in OF and specified the residual weights in the models due to the high variance within individuals from HQ treatment. For the time in the center, differences in temperature in the OF and exploration time and latency to leave trap for NE we used the function lme4::lmer (Pinheiro and Bates 2023). As response variables, we used each of the measurements for OF and NE, whereas treatment, sex, and trials (1 and 2) as well as the interaction treatment:trial for the flexibility experiment were fitted as fixed effects. Individual identity was used as a random effect. Gaussian distribution was assumed for all models except for the number of trips in NE which was assumed to follow a Poisson distribution. Variables and model fit were checked using q–q plots. Latency to leave trap to start exploration and time spent in OF center were square-root transformed to yield a better model fit. For significant effects, we did a post hoc comparison with FDR approach, designed to compare all different combinations of treatment and sex using, lsmeans::lme (Lenth 2016).

We evaluated the repeatability separately by treatments for all measurements from OF and NE using similar data distributions and transformations as above. We used unadjusted (i.e., a model containing no fixed effects other than trial) repeatability at the individual level using rptR::rpt (Stoffel et al. 2017).

#### Life-history

To test if a food-switch has an immediate effect on body mass development, we ran a linear mixed model using the function nlme::lme (Pinheiro and Bates 2023) with body mass change within the month before the food-switch and within 1 month after the food-switch as a response, and treatment, sex, time (before or after) as well as the interaction of treatment:time as fixed and individual ID as random effect. To test if the food quality had an intergenerational effect on body mass development, we ran a linear mixed model using the function nlme::lme (Pinheiro and Bates 2023), the actual body mass was used as response, treatment, and sex and month (as a factor) as fixed and individual ID as a random effect.

To assess the flexibility response in reproduction, we ran generalized linear models using the function stats::glm (R version 4.1.3, R Core Team 2022) to test for treatment differences in the percentage of reproductively active females one month after the food-switch. The average percentage of reproductively active females per month was similarly analyzed including data from months 2–6 for each female for the inter-generational part.

To calculate how a food-switch affects immediate survival, we compared the surviving number of all animals and of males separately during the month preceding the food-switch and after the food-switch using two the function stats::prop.test (R version 4.1.3, R Core Team 2022). To estimate the survival of individuals from one generation after the food-switch, we used the function survival::Surv and survival::survfit (Therneau 2023), which uses the Kaplan–Meier method to calculate the survival curves between treatment and sexes within treatments.

## Results

### Behavior

**Figure 2.**
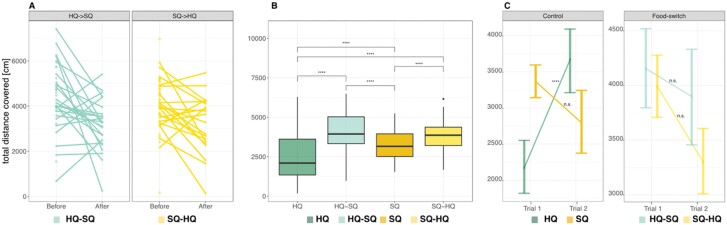
Distance covered in the OF (A) after a food-switch in adulthood, (B) one generation after the food-switch and (C) average of distance covered from trial 1 and trial 2 one generation after the food-switch. (A) Differences in distance covered per individual, before (trial 1) and after (trial 2) the food-switch. Green lines on the left show food-switch from HQ to SQ and yellow on the right food-switch from SQ–HQ. (B) Box plots (averages across both trials) and comparison between food quality treatments, HQ (high quality food), SQ (standard quality food), HQ–SQ food switch from HQ to SQ, HQ–SQ food switch from HQ to SQ. Significant effects for each pair-wise comparison are indicated by “ **** “. (C) Line plot, vertical lines show the variance of the measurements across all individuals per treatment and trial, points represent the average and diagonal lines show the direction of change from trial 1 to trial 2. Significant values are indicated by “ **** “

Comparable to previous results (Prabh et al. 2023), control SQ animals covered on average more distance in OF than control HQ animals (*t* = −3.622; post hoc *P* = 0.001, Figure 2B) but did not differ in any other behavioral trait (Supplementary Information Table 1). Similarly, here we only found the effects of food-switches on the distance covered. Behavior did not adjust according to an adaptive scenario after a food-switch, indicated by non-significant interaction terms between treatment and trial in the within-generation food-switch. However, regardless of the direction of food-switch, all animals reduced the distance covered in the OF directly after the food-switch, that is, in trial 2 of the OF (*t* = −4.633, *P* < 0.001, Figure 2A, Supplementary Information Table 3). Offspring of food-switched parents covered on average more distance in the OF than both control treatments (Figure 2B), independent of the direction of switch (Supplementary Information Table 1 HQ—HQ–SQ: *t* = 7.662; post hoc *P* < 0.001; SQ—SQ–HQ: *t* = 2.852; post hoc *P* = 0.006). Except the HQ control treatment, animals in all other treatments covered similar distance in trials 1 and 2 (estimate = −1544.7, SE = 313, df = 158, *t* = −4.937 post hoc *P* < 0.0001; see Figure 2C).

All behaviors and the temperature difference were significantly repeatable in animals receiving a food-switch in adulthood (Table 2A). In offspring of food-switched parents as well as in control treatments, most variables did not show a significant repeatability with the sample sizes we used here (Table 2B). However, the same behaviors have been shown to be repeatable in a previous study (Prabh et al. 2023) using bigger sample size.

**Table 2.** Repeatability for risk-taking using one forced exploration test OF and one voluntary exploration test (NE), the sample size of individuals tested in trials 1 and 2 is shown in column N. (A) Food-switch during adulthood and (B) Offspring of food-switched animals. Estimates are based on linear mixed models (LMMs). Repeatabilities (R) and 95% confidence intervals (CI) are shown. Significance (P) estimates are based on 1000 random permutations, significant repeatabilities are in bold font.

(A) Repeatability flexibility									
OF	N	Treatment	*R*	P permut	CI	NE	R	P permut	CI
Distance covered	45	HQ–SQ/SQ–HQ	0.218	0.085	0-0.481	Exploration time	**0.375**	0.007	0.05–0.571
ΔT	45	HQ–SQ/SQ–HQ	**0.445**	0.003	0.199- 0.662	Latency to leave trap	**0.344**	0.012	0.078, 0.565
Time in the centre	45	HQ–SQ/SQ–HQ	**0.355**	0.018	0.104- 0.575	Trips	**0.46**	0.007	0.251–0.715
B) Repeatability Intergenerational for OF and NE									
OF	N	Treatment	R	P permut	CI	NE	R	P permut	CI
Distance covered	15	HQ	**0.691**	0.004	0.4–0.891	Exploration time	0.12	0.405	0–0.645
	16	SQ	0	1	0–0.53		**0.716**	0.015	0.375–0.927
	16	HQ–SQ	0.236	0.225	0–0.669		0.581	0.143	0–0.936
	15	SQ–HQ	0.232	0.239	0–0.69		0.367	0.195	0–0.84
ΔT	15	HQ	0	1	0–0.634	Latency to leave trap	0.191	0.326	0–0.679
	16	SQ	0.185	0.324	0–0.671		0.048	0.504	0–0.703
	16	HQ–SQ	0	1	0–0.548		**0.787**	0.021	0.449–0.964
	15	SQ–HQ	0.135	0.377	0–0.641		0	1	0–0.686
Time in the center	15	HQ	0	1	0–0.546	Trips	0	1	0–0.278
	16	SQ	0	0-0.543	1		0	1	0–0.278
	16	HQ–SQ	0	1	0–0.557		0.303	0.235	0–0.51
	15	SQ–HQ	0.124	0.382	0–0.625		0.154	0.309	0–0.359

We also assessed differences between males and females, however, sex was never a significant predictor.

### Life-history

**Figure 3.**
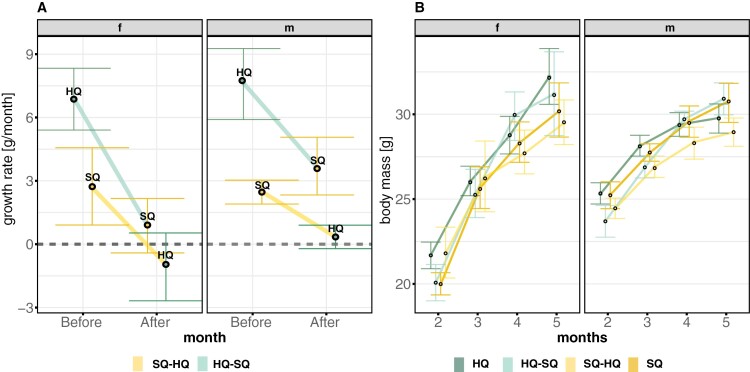
Effect of food-switch on the mean of (A) growth rate one month before versus one month after a food-switch and (B) body mass development one generation after a food-switch. (A) The error bars are around the mean (black point) of the variation in the growth rate on the current treatment, (HQ green and SQ yellow) before and after the food switch, the connecting lines show the direction of the switch (from HQ to SQ light green and from SQ to HQ light yellow). Females are shown left and males right. (B) The error bars are around the mean (black points) of the variation in body mass per month, the left side shows body mass change in females and right in males.

Growth rates before the food-switch in adulthood were higher in HQ compared with SQ animals (Figure 3A). The food-switch affected SQ and HQ animals differently. Although HQ animals grew on average 7.190 g in the month before the food switch to SQ, they only 1.794 g in the month after the food-switch, thus reducing growth by about 4 times after the food-switch (see Supplementary Information Table 5, treatment: time: *F*-value = 16.224, Pr(>*F*) < 0.001). SQ animals grew 2.581 g in the month before the food-switch and reduced growth even more strongly (*t* = 2.535, post hoc *P* = 0.0186, Figure 3A) to 0.433 g in the month after the food-switch. Descendants of animals that received a food-switch from HQ to SQ initially weighed less than the control HQ (*t* = 2.535, post hoc *P* = 0.0186) but were similar to SQ. After weaning, HQ–SQ animals did not differ significantly anymore from pure HQ animals and also did not differ significantly from pure SQ animals, that is, their growth rates were intermediate between the two control treatments (Figure 3B). Individuals whose parents were switched from SQ to HQ started with similar body masses as control SQ but tended to weigh less than control SQ in month 5 (*t* = −2.137, post hoc *P* = 0.066).

In addition, there was a strong sex effect. Sons but not daughters from food-switched SQ-HQ differed from SQ (*t* = −3.294, *P* = 0.0040), SQ–HQ males also tended to differ from HQ males (*t* = 2.210, *P* = 0.084), indicating a stronger effect of the parental food-switch in males.

**Figure 4.**
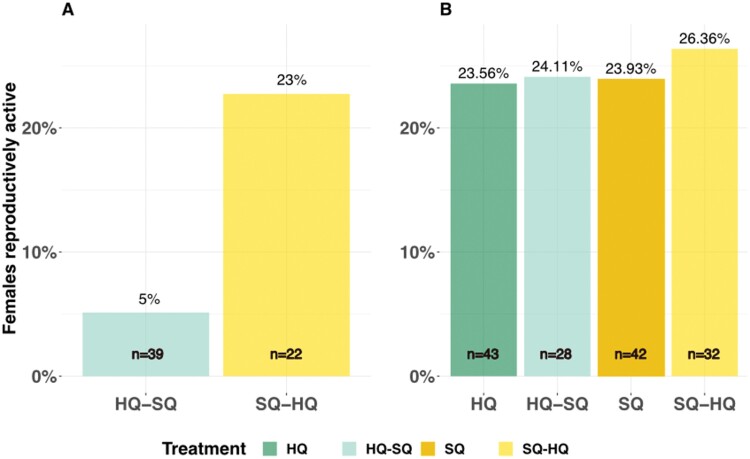
Percentage of reproductively active females (A) one month after a food-switch and (B) one generation later. The percentages were calculated as number of reproductively active females out of all adult (age ≥ 3 months) in the population (A) after the food switch (B) in the F4 generation averaged across months. The percentages are displayed on top of the bars and the sample sizes are shown on the bottom of the bars.

Although on average between 20% and 30% of females were reproductively active in control treatments and in first-generation descendants of food-switched animals during any monitoring (Figure 4B), only 5% of females were reproductively active (*z* = 1.911, *P* = 0.05), 1 month after a food-switch from HQ–SQ but a switch from SQ–HQ did not affect the percentage of reproductively active females (Figure 4A).

**Figure 5.**
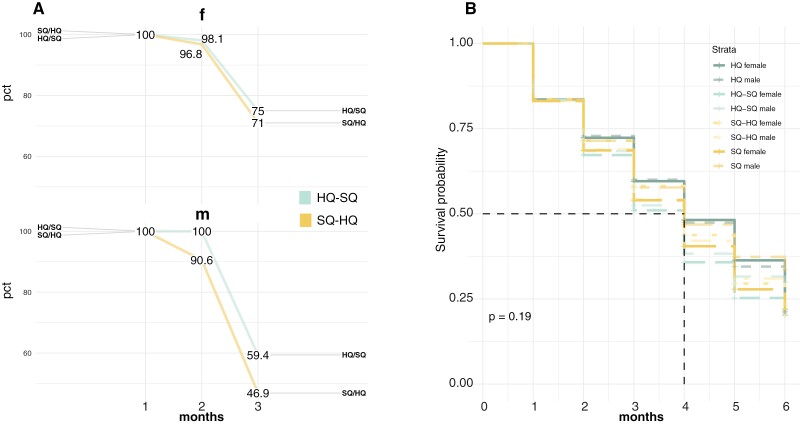
Percentage of survival before and after food-switch in adulthood (A) and intergenerational (B). (A) Slope graph, in animals that experienced a food-switch in adulthood: the lines show the percentage of the individual survival, and the *x*-axis shows the monthly monitoring (i.e., 1—before the food-switch, 2—the day of the food-switch and 3—after the food-switch). (B) Survival intergenerational: Kaplan–Meier survival estimate: the graphs show the proportion of individuals surviving in each treatment during 6 months. The 8 lines represent females and males for each treatment, a vertical drop in the curve indicates an event in the percentage of surviving individuals. The horizontal dashed line is a median survival.

Survival rates decreased with age for both sexes but independently from food quality or whether or not experiencing a food-switch directly or in the parental generation (Figure 5; Supplementary Information Tables 7and 12).

## Discussion

**Figure 6.**
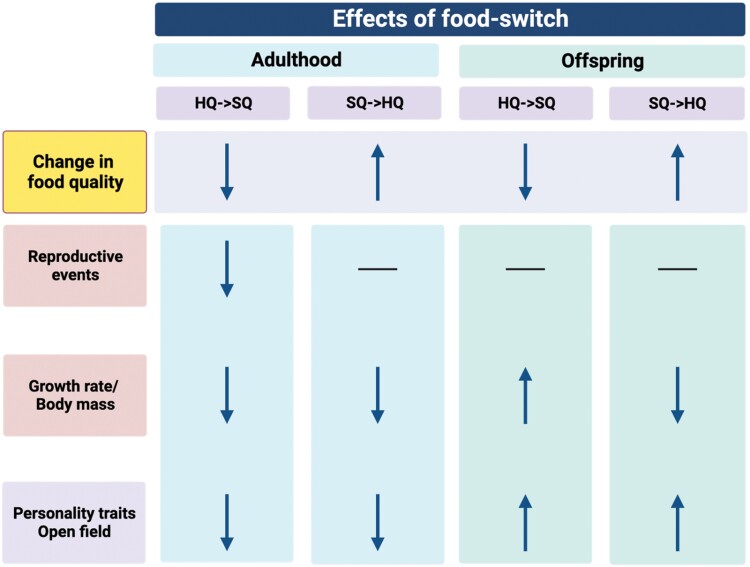
Graphical summary. The effects of food-switch in the flexibility (adulthood) and intergenerational (offspring) experiments. Downwards arrows indicate reduction, upwards arrows indicate increase and horizontal lines indicate no change in the traits or environmental factors shown on the left.

We conducted this study to examine how fast animals can adjust their behavioral and life-history phenotype to an environmental change and whether the strength or speed of adjustment would be condition-dependent in which case HQ animals would have an advantage. Specifically, we investigated the impacts of food-switches during 1) adulthood, that is, potential within-generation changes and (2) across generations on the responses of wild house mice (*Mus musculus*) (see Figure 1). Our findings (graphically summarized in Figure 6), revealed that individuals who experienced a food-switch during adulthood did not adjust their behavior immediately in an expected way, that is, changing their behavior in the direction of the corresponding control treatment mean but rather they all became more passive stress-copers. Offspring that grew up with different food quality than their parents exhibited a more active stress-coping response, that is, covering more distance in the OF than offspring of both control populations independent of the direction of the food-switch. This indicates a general overshoot (i.e., the magnitude of change being larger than the difference between the starting and the goal treatment, i.e., describing a process) and therefore constitutes a mismatch response (i.e., the behavioral expression not matching the respective control treatments expression, that is, describing the outcome of a process) (see Figure 2). In contrast, life-history traits, that is, body mass development and reproduction but not survival (see Figures 3–5), reacted immediately after a food-switch and only effects on body mass development persisted into the next generation (Figure 3B). Although offspring of animals that were switched from worse to better food showed intermediate growth rates, animals that were switched from better to worse, showed a mismatch pattern, ending up with lower adult body masses than their respective control population (Figure 3B). For all types of traits, we observed that animals that switched from a better to a worse-quality diet exhibited stronger behavioral and life-history responses compared with those that switched in the opposite direction.

Under an adaptive scenario, we expected animals being switched from worse to better conditions (within or across generations) to adjust their stress-coping style to a more passive strategy, whereas animals being switched from better to worse conditions should develop a more active stress-coping strategy. Within one month after a food-switch, this did not happen. Animals in both treatments reduced the distance covered and became less active stress-copers in the second trial (Figure 2A), whereas the offspring treatments generally did not change their behavior between the two trials (except the HQ treatment, Figure 2C). The animals’ behavior changing in a consistent direction after an environmental change, irrespective of the direction of the food-switch, shows that the animals were not responding to the shift in quality, but to the change itself. A similar reduction in risk-taking behaviors (emergence latency to leave trap and exploration activity) was found in common voles (*Microtus arvalis*) when animals were translocated from their natural range to a laboratory setting (Mazza et al. 2020). The magnitude of change in this study depended on whether the source environment of the animals was rural or urban (Mazza et al. 2020), with urban animals showing a higher degree of flexible adjustment. In the context of urbanization and adjusting to other novel/changing environments, such a reduction in risk-taking may thus be an adaptive mechanism that small mammals have evolved in the past.

For the SQ and HQ control treatments, we found SQ animals to be more active stress-copers (Figure 2B) as was already reported in previous generations (Prabh et al. 2023). Offspring whose parents experienced a food-switch were more active in the OF than those who grew up in conditions that matched their parents’ conditions, corresponding to the match-mismatch hypothesis (Figure 2B). Other studies in the past have found mismatching offspring behaviors, for example, Krause and Naguib (2014). Zebra finches (*Taenophygia guttata*) growing up under different food qualities than their parents developed intermediate exploration behavior (Krause and Naguib, 2014). In snails (*Physa acuta*), offspring experiencing a mismatching predation regime than their parents, likewise showed an intermediate escape behavior (Tariel et al. 2020). These and other studies in the past interpreted their findings in terms of the match-mismatch hypothesis without testing possible effects on fitness. Here, we provide results on the immediate fitness and fitness of the offspring generation. Although immediate fitness was reduced when switching from better to worse food (Figure 4A), no effects were found when switching from worse to better or in the offspring generation. Interestingly, both previously published examples (Krause and Naguib 2014; Tariel et al. 2020) found the offspring to already adjust their phenotype into the direction of the new treatment optimum (as expressed by the respective control group) but expressing an intermediate mismatching phenotype because the magnitude of change was not large enough to reach the new optimum yet. We on the other hand, find a mismatch, i.e., expressing a more active stress-coping strategy than both control populations in the mismatching scenarios and an overshoot, that is, the magnitude of change exceeding the magnitude that would have been necessary to reach the new treatment mean (Figure 2B). Mathematical models suggested such an overshoot to be likely when the environmental change is too strong for parental effects to produce optimal phenotypes (Hoyle and Ezard 2012). Likewise, parental effects are only expected to shape offspring phenotypes optimally if the environmental change is predictable, i.e., if parents know how to react to the environmental cue (Uller 2008; Burgess and Marshall 2014). Because in our experiment the environment had been stable for three generations before the switch, it may have constituted an unexpected environmental change and elicited a general, first-line response irrespective of the direction of food-switch.

Alongside effects of a parental food-switch on mean-level behavioral expressions, we also found effects on the repeatability of behavioral traits. Although adults from both treatments in which a food-switch was conducted in adulthood showed significant repeatability (Table 2), in the descendants after the food-switch, repeatability was treatment- and trait-specific. This was unexpected because previous results on the same study system albeit with a larger sample size (Prabh et al. 2023), showed repeatability for both HQ and SQ treatments. Previous studies in field crickets (*Gryllus bimaculatus*), showed that individuals experiencing a diet high in protein behaved less stably (i.e., have higher within-individual variance), showing that food quality can influence individual differences, not just in average behavior, but also in variance (Han and Dingemanse 2017).

Although behavioral traits did not adjust adaptively to a food-switch immediately within 1 month, a food-switch immediately influenced reproduction and body mass development (Figures 3A–4A). Females being switched from better to worse strongly reduced reproduction whereas this did not happen in females switching from worse to better food. A similar result was found in a long-term experiment in the cichlid *S. pleurospilus*, where females switched from better to worse reproduced slowly with smaller clutch sizes than females that were switched from worse to better (Taborsky 2006). In our study, this effect on reproduction did not persist into the next generation, in which females across all treatments reproduced comparably.

A higher percentage of males died after the food-switch compared with females; independent of the direction of the food-switch, (Figure 5). This is an indication that male house mice may be more sensitive to environmental change compared with females. In fish and some bird species, sex-biased survival according to environmental conditions and especially after an environmental change, is well-known (Geffroy et al. 2020). Often, males suffer more from that is, increased temperatures. This has not yet been described to our knowledge for mammals and needs further investigations.

A food-switch in adulthood reduced growth (HQ–SQ) or stopped growth completely and even induced a slight weight loss (SQ–HQ) (Figure 3A). Surprisingly, animals could not adjust quickly to the higher nutrient content and energy availability of the better diet, potentially again indicating an unpredictable environmental change or a general response toward novel environmental conditions. The simplest explanation would be that animals consumed less food due to an avoidance reaction. However, in the past, we found that mice generally preferred a high-quality diet if given the chance to select (Prabh et al. 2023). Alternatively, the mechanism of switching from a worse to a better-quality food might differ and take longer to adjust than vice-versa. It has been shown that access to protein-rich diets ad libitum, promotes and maintains weight loss, and previous studies suggest that at least part of this effect resulted from reduced total energy intake (Sørensen et al. 2008; Huang et al. 2013). High protein intake has also been shown to induce sustained reductions in appetite, ad libitum caloric intake, and body weight (Sørensen et al. 2008; Huang et al. 2013). Another explanation might be related to the observed effects on reproduction. A shift from better to worse food might induce an “emergency life-history stage” triggering animals to stop reproduction (Wingfield et al. 1998; Pahuja and Narayan 2021) as we found here. Hence, they may be better able to preserve their body mass than animals which did not stop reproducing.

Growth in offspring of food-switched parents strongly depended on the direction of the food-switch (Figure 3A). Offspring of parents that were switched from worse to better had an advantage at weaning since their body mass at that time did not differ from pure HQ animals (i.e., a silver-spoon effect). This suggests that their mothers were able to promote high growth despite experiencing a food-switch and continuing reproduction immediately. Offspring of parents being switched from better to worse were weaned with similar body masses as SQ animals, suggesting that their mothers were not able to promote their growth despite stopping reproduction for a certain time after a food-switch (Figure 4A). After weaning, offspring of mice being switched from better to worse showed intermediate growth between pure HQ and SQ after independence (Figure 3B), suggesting some lasting benefits from their parents. On the other hand, offspring of parents switching from worse to better, showed the least growth despite the initial boost until weaning (Figure 3B).

Our experiments support a strong condition-dependence of food-switch on life-history traits (both, immediate, and carried-over to offspring, Figure 6) and on stress-coping behavior. It is already well known that risk-taking behaviors are likely condition-dependent. Although poor nutritional conditions can promote high risk-taking behaviors in animals (Royauté and Dochtermann 2017; Beyts et al. 2020), animals in good nutritional condition may be less likely to engage in risky behaviors because they have access to the resources they need to survive and reproduce (English et al. 2016; Barclay et al. 2018; Moran et al. 2021). In offspring of animals switching from better to worse, the increase in the distance covered in OF was nearly three times stronger compared with the observed increase in offspring of animals switching from worse to better which resulted in similar behavioral expressions of mean level-behaviors. For life history traits, we observed that a switch from worse to better affected mainly body mass development whereas strongest effects were observed on reproduction when switching from better to worse. Although switching from worse to better strongly reduced growth, reproductive events did not collapse. This is in contrast to a shift from better to worse, which caused a near-complete stop of reproduction.

## Conclusion

In summary, both, behavior and life history, reacted towards a change in diet, at first, with an unspecific change not directed towards the new treatment optimal phenotypic expression. Behavioral traits need more than one generation to adjust to a (potentially unexpected) change in food quality adaptively. Although survival here was unaffected and the probability to reproduce only showed a short-term reduction in one treatment but no carry-over effects to the next generation, growth was immediately reduced and, similarly to behavior, did not adjust adaptively within one generation. For behavioral and life-history traits, we observed a strong condition-dependence with animals previously fed on HQ food regularly showing stronger changes after a change of environment. Such condition-dependence may influence the ability of animals to adjust to environmental change and should therefore be taken into account, for example, high-quality nutritional environments during development might enable individuals to more flexibly respond to micro-environmental variation experienced during adulthood.

## Supplementary Material

zoae005_suppl_Supplementary_Material
