# Supplementary material for: Is the speed of adjusting to environmental change condition dependent? An experiment with house mice (Mus musculus)
Source: Curr Zool. 2024 Mar 7;70(3):350–60. doi: 10.1093/cz/zoae005 (PMC11256001; doi:10.1093/cz/zoae005)
Supplement: zoae005_suppl_Supplementary_Material [file zoae005_suppl_supplementary_material.docx]

**Supplementary Information for: Is the speed of adjusting to environmental change condition dependent? An experiment with house mice (*Mus musculus*)**

^1^Karem Lopez-Hervas, ^1,2^Neelam Porwal, ^1,3,4^Mathilde Delacoux, ^1,5^Alexandros Vezyrakis, ^1^Anja Guenther

Karem Lopez-Hervas

Email: [lopezhervas@evolbio.mpg.de](mailto:lopezhervas@evolbio.mpg.de)

1RG Behavioural Ecology of individual differences, Max Planck Institute for Evolutionary Biology, 24306 Plön, Germany

2 Department of Evolutionary Biology, Faculty of Biology, Adam Mickiewicz University, Wieniawskiego 1, 61-712 Poznań, Poland

3 Department for Collective Behaviour, Max Planck Institute of Animal Behaviour, 78464 Constance, Germany

4 Centre for the Advanced Study of Collective Behaviour, University of Konstanz, 78464 Constance, Germany

5 Animal Ecology, Institute for Biochemistry and Biology, University of Potsdam, 14469

Potsdam, Germany

| **Table 1.** Post-hoc comparison for risk-taking using a forced exploration test (Open Field, left column) and a voluntary exploration test (Novel environment, right column). Pairwise comparisons between offspring whose parents experienced a food switch (HQ-SQ and SQ-HQ) and control treatments (HQ and SQ, i.e., no food-switch). T-values and p-values of Linear Mixed Models are reported for each comparison, with significant values in bold. | | | | | | | | | | | |
| --- | --- | --- | --- | --- | --- | --- | --- | --- | --- | --- | --- |
|  | **Variables Open Field** | | | | **Variables Novel Environment** | | | | | | |
|  |  | **T-value** | **P-value** | |  | | **T-value** | **P-value** | | |  |
| HQ vs SQ | Distance covered | **-3.622** | | **<.001** | Latency to leave trap | 0.105 | | | 0.917 |  |  |
|  | ΔT | 1.087 | | 0.556 | Exploration time | -0.430 | | | 0.822 |  |  |
|  | Time spent in the center | 0.870 | | 0.596 | Trips | 0.329 | | | 0.916 |  |  |
| HQ vs HQ-SQ | Distance covered | **-7.662** | | **<.001** | Latency to leave trap | 0.372 | | | 0.916 |  |  |
|  | ΔT | 0.864 | | 0.583 | Exploration time | 0.225 | | | 0.822 |  |  |
|  | Time spent in the center | 1.690 | | 0.450 | Trips | 1.081 | | | 0.908 |  |  |
| HQ vs SQ-HQ | Distance covered | **-6.343** | | **<.001** | Latency to leave trap | 0.535 | | | 0.917 |  |  |
|  | ΔT | -0.649 | | 0.620 | Exploration time | -0.660 | | | 0.822 |  |  |
|  | Time spent in the center | 0.185 | | 0.853 | Trips | 0.106 | | | 0.916 |  |  |
| SQ vs SQ-HQ | Distance covered | **2.852** | | **0.006** | Latency to leave trap | -0.424 | | | 0.917 |  |  |
|  | ΔT | 1.735 | | 0.419 | Exploration time | 0.230 | | | 0.822 |  |  |
|  | Time spent in the center | 0.644 | | 0.594 | Trips | 0.223 | | | 0.916 |  |  |
| SQ vs HQ-SQ | Distance covered | **4.150** | | **0.001** | Latency to leave trap | -0.266 | | | 0.917 |  |  |
|  | ΔT | 0.189 | | 0.850 | Exploration time | -0.648 | | | 0.822 |  |  |
|  | Time spent in the center | -0.805 | | 0.594 | Trips | -0.749 | | | 0.916 |  |  |
| HQ-SQ vs SQ-HQ | Distance covered | 1.244 | | 0.215 | Latency to leave trap | 0.147 | | | 0.917 |  |  |
|  | ΔT | -1.483 | | 0.420 | Exploration time | -0.878 | | | 0.822 |  |  |
|  | Time spent in the center | -1.439 | | 0.456 | Trips | -0.977 | | | 0.908 |  |  |

| **Table 2.**  Post-hoc comparison for risk-taking using a forced exploration test (Open Field, left column) and a voluntary exploration test (Novel environment, right column). Comparison between individuals that experienced a food-switch during their lifetime before (Trial 1) and after (Trial 2) the food switch. T-values and p-values of Linear Mixed Models are reported for each comparison, with significant values in bold. | | | | | | |
| --- | --- | --- | --- | --- | --- | --- |
|  | **Variables Open Field** | | | **Variables Novel Environment** | | |
|  |  | **T-value** | **P-value** |  | **T-value** | **P-value** |
| HQ-SQ Trial 1  vs  SQ-HQ Trial 1 | Distance covered | 1.020 | 0.371 | Latency to leave trap | -0.158 | 0.874 |
|  | ΔT | 0.221 | 0.825 | Exploration time | 0.722 | 0.566 |
|  | Time spent in the center | -0.130 | 0.896 | Trips | -0.435 | 0.663 |
| HQ-SQ Trial 2 vs  SQ-HQ Trial 2 | Distance covered | -0.180 | 0.857 | Latency to leave trap | 1.357 | 0.212 |
|  | ΔT | 0.843 | 0.481 | Exploration time | -0.499 | 0.618 |
|  | Time spent in the center | 0.340 | 0.881 | Trips | -1.468 | 0.196 |
| HQ-SQ Trial 1  vs  HQ-SQ Trial 2 | Distance covered | **3.882** | **0.001** | Latency to leave trap | **2.638** | **0.021** |
|  | ΔT | 2.131 | 0.074 | Exploration time | -1.583 | 0.178 |
|  | Time spent in the center | -0.967 | 0.881 | Trips | **-2.492** | **0.025** |
| SQ-HQ Trial 1  vs  SQ-HQ Trial 2 | Distance covered | **2.605** | **0.016** | Latency to leave trap | **4.524** | **0.000** |
|  | ΔT | **2.930** | **0.027** | Exploration time | **-2.966** | **0.025** |
|  | Time spent in the center | -0.360 | 0.881 | Trips | **-4.252** | **0.000** |

| **Table 3.** ANOVA-output of linear mixed models for a forced exploration test (Open Field) in individuals that experienced a food-switch during their lifetime. | | | | | | | | |  |
| --- | --- | --- | --- | --- | --- | --- | --- | --- | --- |
|  | **Sum Sq** | **Mean Sq** | **Numdf** | | | **Dendf** | **F value** | **Pr(>F)** | |
| **Temperature differences** | | | | | | | | |  |
| Treatment | 0.129 | 0.129 | | 1 | 83.870 | | 0.442 | 0.508 | |
| Sex | 0.322 | 0.322 | | 1 | 79.340 | | 1.104 | 0.297 | |
| Trial | 3.879 | 3.879 | | 1 | 62.320 | | **13.290** | **0.001** | |
| Treatment:Trial | 0.128 | 0.128 | | 1 | 62.430 | | 0.438 | 0.510 | |
| **Distance covered** | | | | | | | | |  |
| Treatment | 233309 | 233309 | | 1 | 78.160 | | 0.180 | 0.673 | |
| Sex | 6098 | 6098 | | 1 | 76.850 | | 0.005 | 0.946 | |
| Trial | 27713495 | 27713495 | | 1 | 71.620 | | **21.360** | **<0.001** | |
| Treatment:Trial | 896706 | 896706 | | 1 | 71.830 | | 0.691 | 0.409 | |
| **Time spent in the centre** | | | | | | | | |  |
| Treatment | 1.603 | 1.603 | | 1 | 81.230 | | 0.021 | 0.885 | |
| Sex | 44.400 | 44.400 | | 1 | 77.610 | | 0.580 | 0.449 | |
| Trial | 65.310 | 65.310 | | 1 | 64.610 | | 0.853 | 0.359 | |
| Treatment:Trial | 13.950 | 13.950 | | 1 | 64.760 | | 0.182 | 0.671 | |

| **Table 4.a** ANOVA-output of a Linear Mixed Model for a voluntary exploration test (NE) in latency to leave trap and exploration time in individuals that experienced a food-switch during their lifetime. | | | | | | | | | | | | | |
| --- | --- | --- | --- | --- | --- | --- | --- | --- | --- | --- | --- | --- | --- |
|  | **Sum Sq** | **Mean Sq** | **Numdf** | | **Dendf** | | **F value** | | | | **Pr(>F)** | | |
| **Latency to leave trap** | | | | | | | | | | | | | |
| Treatment | 35.120 | 35.120 | 1 | 71.380 | | | 0.959 | | 0.330 | | | |  |
| Sex | 69.100 | 69.100 | 1 | 67.620 | | | 1.888 | | | 0.174 | | |  |
| Trial | 949.900 | 949.900 | 1 | 56.190 | | | **25.950** | | | **<0.001** | | |  |
| Treatment:Trial | 66.420 | 66.420 | 1 | 56.250 | | | 1.814 | | | 0.183 | | |  |
| **Exploration time** | | | | | | | | | | | | | |
| Treatment | 17348 | 17348 | 1 | 65.220 | | | 1.067 | | | | 0.305 | |  |
| Sex | 32158 | 32158 | 1 | 61.480 | | | 1.978 | | | | 0.164 | |  |
| Trial | 176408 | 176408 | 1 | 48.470 | | | **10.850** | | | | **<0.001** | |  |
| Treatment:Trial | 16461 | 16461 | 1 | 48.530 | | | 1.012 | | | | 0.319 | |  |
| **Table 4.b** ANOVA-output of a Generalised Linear Mixed Model for voluntary exploration test (NE) in number of trips measured in individuals that experienced a food-switch during their lifetime. | | | | | | | | | | | | | |
|  | | | | | | | | | | | | | |
|  | **Chisq** | **Df** | **Pr (>Chisq)** | | |  | |  | | | |  |  |
| **Number of trips** | | | | | | | | | | | |  |  |
| Treatment | 1.604 | 1 | 0.293 | | |  |  |  |  |  |  |  |  |
| Sex | 23.143 | 1 | **<0.001** | | |  |  |  |  |  |  |  |  |
| Trial | 9.550 | 1 | **0.002** | | |  |  |  |  |  |  |  |  |
| Treatment:Trial | 1.176 | 1 | 0.278 | | |  |  |  |  |  |  |  |  |

| **Table 5.** ANOVA-output of a Linear Mixed Model for growth rate (g/month) in individuals that experienced a food-switch during their lifetime. | | | | | | | |
| --- | --- | --- | --- | --- | --- | --- | --- |
|  | **Sum Sq** | **Mean Sq** | **Numdf** | **Dendf** | **F value** | **Pr(>F)** |  |
| Treatment | 0.528 | 0.528 | 1 | 231 | **30.216** | **<0.001** |  |
| Sex | 0.006 | 0.006 | 1 | 231 | 0.334 | 0.564 |  |
| Month | 1.064 | 1.064 | 1 | 231 | **60.920** | **<0.001** |  |
| Sex:Month | 0.013 | 0.013 | 1 | 231 | 0.772 | 0.380 |  |
| Treatment:Month | 0.283 | 0.283 | 1 | 231 | **16.224** | **<0.001** |  |

| **Table. 6** Post-hoc comparisons for growth rates in F4 individuals among different treatments before and after food-switch. Each comparison shows the mean growth rate, the standard error (SE), and the corresponding p-value from the Linear Mixed Model. The p-values in bold font indicate significant differences between the compared groups. | | | | | |
| --- | --- | --- | --- | --- | --- |
| **Contrast** | **Estimate** | **SE** | **Df** | **T-value** | **P-value** |
| (HQ-SQ Before) - (SQ-HQ Before) | 0.193 | 0.022 | 231 | **8.558** | **<0.001** |
| (HQ-SQ Before) - (HQ-SQ After) | 0.215 | 0.023 | 128.1 | **9.233** | **<0.001** |
| (HQ-SQ Before) - (SQ-HQ After) | 0.264 | 0.026 | 231 | **9.992** | **<0.001** |
| (SQ-HQ Before) - (HQ-SQ After) | 0.021 | 0.025 | 231 | 0.872 | 0.384 |
| (SQ-HQ Before) - (SQ-HQ After) | 0.070 | 0.028 | 133.3 | **2.535** | **0.018** |
| (HQ-SQ After) - (SQ-HQ After) | 0.049 | 0.028 | 231 | 1.750 | 0.098 |

| **Table 7.**  Survival percentage differences in F4 individuals that experienced the food-switch during adulthood in the two different experimental treatments. The survival percentage difference indicates the percentage change in survival before versus after food-switch in females and males. Estimates were based on a two-proportions z-test. | |
| --- | --- |
| **Survival in females:** X-squared = 1.255 df = 1 p-value = 0.262 | |
| **Prop HQ-SQ females** | **Prop SQ-HQ females** |
| 0.690 | 0.587 |
| **Survival in males:** X-squared = 0.564 df = 1 p-value = 0.452 | |
| **Prop HQ-SQ males** | **Prop SQ-HQ males** |
| 0.593 | 0.469 |

| **Table 8.**  ANOVA of Linear Mixed Model for growth rate in F4 flexibility for individuals that experienced the food switch during adulthood. | | | | | | | |
| --- | --- | --- | --- | --- | --- | --- | --- |
|  | **Sum Sq** | **Mean Sq** | **Numdf** | **Dendf** | **F value** | **Pr(>F)** |  |
| Treatment | 0.528 | 0.528 | 1 | 231 | **30.216** | **<0.001** |  |
| Sex | 0.006 | 0.006 | 1 | 231 | 0.334 | 0.564 |  |
| Month | 1.064 | 1.064 | 1 | 231 | **60.920** | **<0.001** |  |
| Sex:Month | 0.013 | 0.013 | 1 | 231 | 0.772 | 0.380 |  |
| Treatment:Month | 0.283 | 0.283 | 1 | 231 | **16.224** | **<0.001** |  |

| **Table 9.** Output of the Generalised linear model for reproductively active females that experienced the food switch during adulthood | | | |
| --- | --- | --- | --- |
| **Predictors** | **Odds Ratios** | **CI** | **P-value** |
| (Intercept) | 0.05 | 0.01 – 0.18 | **<0.001** |
| Treatment (SQ-HQ) | 5.44 | 1.06 – 40.75 | 0.056 |
| Observations | 61 | | |
| R^2^ Tjur | 0.070 | | |

| **Table 10.** ANOVA of the Linear Mixed Model for body mass for offspring of food-switched animals | | | | | | |
| --- | --- | --- | --- | --- | --- | --- |
|  | **Sum SQ** | **Mean Sq** | **Numdf** | **Dendf** | **F value** | **Pr(>F)** |
| Treatment | 25.5 | 8.52 | 3 | 334.49 | 1.5343 | 0.2054 |
| Month | 9235.7 | 3078.57 | 3 | 1036.2 | **554.686** | **<0.001** |
| Sex | 168.3 | 168.3 | 1 | 334.25 | **30.323** | **<0.001** |
| Treatment:(Month) | 266.9 | 29.66 | 9 | 1036.66 | **5.3434** | **<0.001** |

| Table 11. Generalised linear model output for reproductively active females of F4 who are offspring of food-switched or control animals. Significant p-values reported in bold. | | | |
| --- | --- | --- | --- |
| Predictors | **Odds Ratios** | **CI** | **P-value** |
| (Intercept) (HQ) | 0.31 | 0.21 – 0.43 | **<0.001** |
| Treatment (HQ-SQ) | 1.03 | 0.59 – 1.79 | 0.916 |
| Treatment (SQ-HQ) | 1.16 | 0.68 – 1.96 | 0.578 |
| Treatment (SQ) | 1.02 | 0.62 – 1.69 | 0.938 |
| Observations | 578 |  |  |
| R^2^ tjur | 0.001 |  |  |

| **Table 12.** Cox proportional hazards regression model of the survival of offspring of food-switched animals. | | | |
| --- | --- | --- | --- |
| **Predictors** | **Estimates** | **CI** | **P-value** |
| Treatment [HQ-SQ] | 1.11 | 0.96 – 1.29 | 0.152 |
| Treatment [SQ-HQ] | 1.09 | 0.95 – 1.24 | 0.227 |
| Treatment [SQ] | 1.00 | 0.88 – 1.14 | 0.973 |
| Sex [m] | 0.92 | 0.83 – 1.01 | 0.088 |
| Observations | 2410 | | |
| R^2^ Nagelkerke | 0.003 | | |

**Open Field**

**
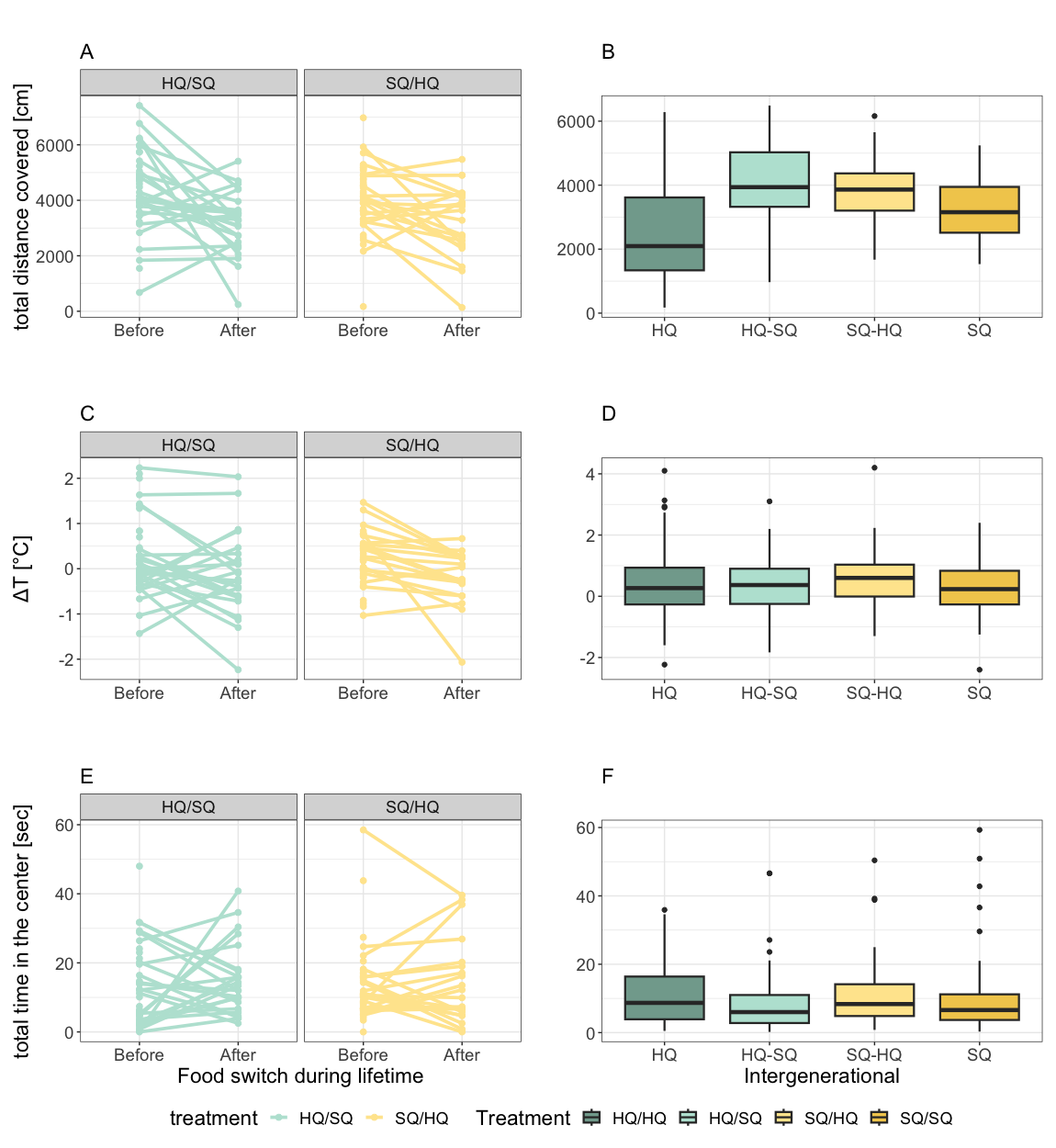
**

n=37

n=44

n=44

n=37

n=37

n=44

n=49

n=41

n=41

n=46

n=49

n=41

n=41

n=51

n=49

n=41

n=41

n=51

Figure. S1 Effect of food-switch on risk-taking behaviour using forced exploration test Open Field (OF). The left panel shows the response of individuals that experienced the food-switch during adulthood, and the right panel shows the response of the offspring generation. A-B) distance covered, the total distances in cm that mice covered in OF, C-D) differences in temperature in °C (before and after of OF test), E-F) time in seconds that mice spent in the centre during the OF test. The lines connecting the paired data points indicate the change following the food-switch.

**Novel environment**


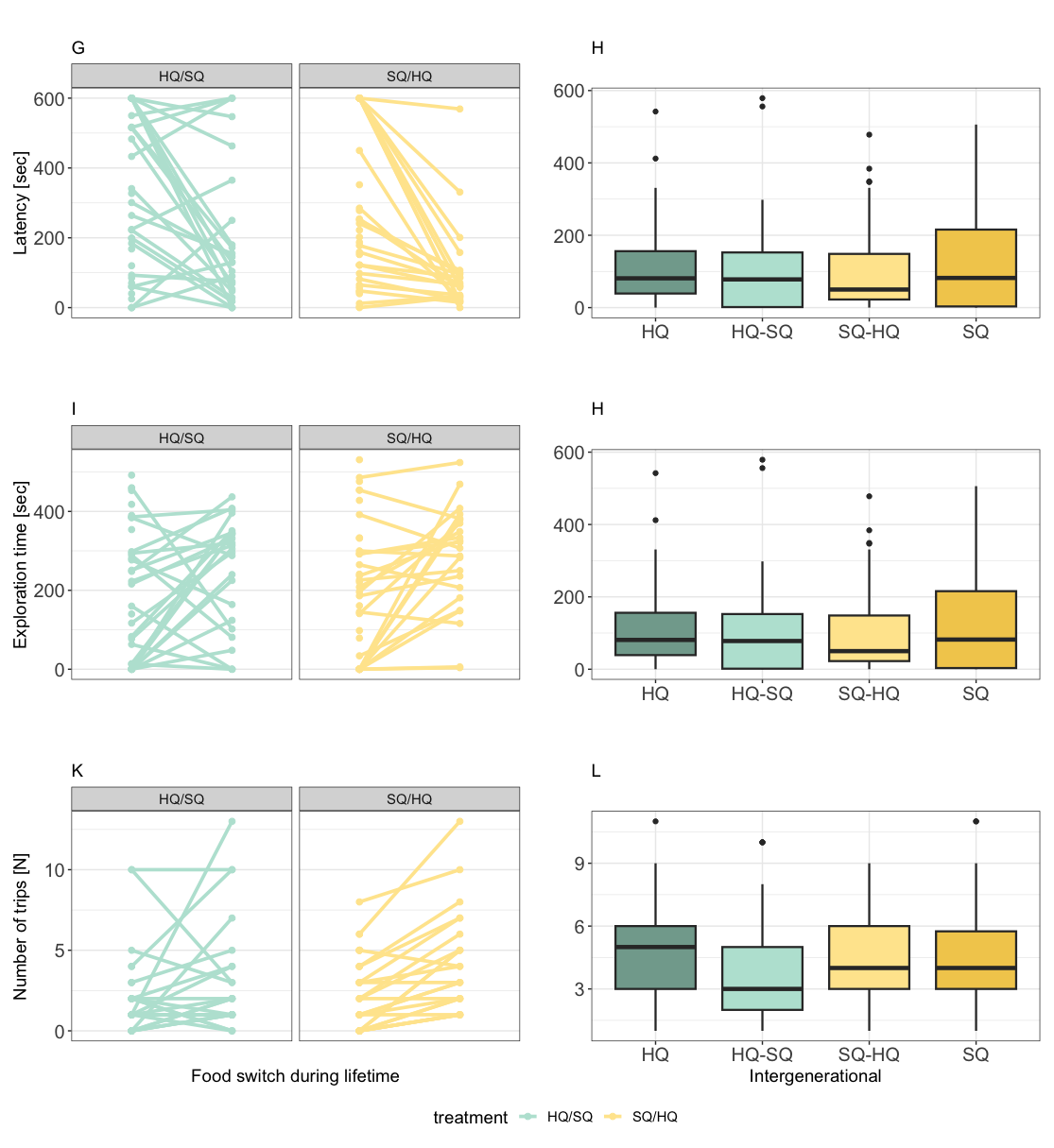


H

G

Figure. S2 Effect of food-switch in risk-taking behaviour using the voluntary exploration test, Novel environment (NE). The left panel shows the response of individuals that experienced the food-switch during adulthood, and the right panel shows the response of the offspring from food-switched animals. G-H) Latency to leave trap, the time in seconds that each mouse spent inside the trap before leaving it to explore the NE, I-J) Exploration time, the time in seconds that each mouse spent actively exploring the NE, K-L) Number of trips, the number of trips that each mouse performed during the NE. The lines connecting the paired data points indicate the change following the food-switch.

n=51

I

K

L

M

n=23

n=24z

n=24

n=23

n=24

n=23

n=46

n=51

n=43

n=50

n=50

n=43

n=46

n=46

n=51

n=43

n=50
